# Dual Redox Targeting by Pyrroloformamide A and Silver Ions Enhances Antibacterial and Anti-Biofilm Activity Against Carbapenem-Resistant *Klebsiella pneumoniae*

**DOI:** 10.3390/antibiotics14070640

**Published:** 2025-06-23

**Authors:** Enhe Bai, Qingwen Tan, Xiong Yi, Jianghui Yao, Yanwen Duan, Yong Huang

**Affiliations:** 1 Xiangya International Academy of Translational Medicine, Central South University, Changsha 410013, China; baienhe@csu.edu.cn (E.B.); tanqw220401@wmu.edu.cn (Q.T.); yixin950818@wmu.edu.cn (X.Y.); 207501001y@hunnu.edu.cn (J.Y.); 2 Hunan Engineering Research Center of Combinatorial Biosynthesis and Natural Product Drug Discovery, Changsha 410013, China; 3 National Engineering Research Center of Combinatorial Biosynthesis for Drug Discovery, Changsha 410013, China; 4 Hefei Comprehensive National Science Center, Institute of Health and Medicine, Hefei 230093, China

**Keywords:** pyrroloformamide A, AgNO_3_, carbapenem-resistant *Klebsiella pneumoniae*, redox homeostasis, glutathione, thioredoxin

## Abstract

**Background:** Dithiolopyrrolones (DTPs), such as holomycin and thiolutin, exhibit potent antibacterial activities. DTPs contain a disulfide within a unique bicyclic scaffold, which may chelate metal ions and disrupt metal-dependent cellular processes once the disulfide is reductively transformed to thiols. However, the contribution of the intrinsic redox mechanism of DTPs to their antibacterial activity remains unclear. Herein we used pyrroloformamide (Pyf) A, a DTP with a unique formyl substituent, as a prototype to study the antibacterial potential and mechanism against ESKAPE pathogens, in particular carbapenem-resistant *Klebsiella pneumoniae* (CRKP). **Methods:** The antibacterial and anti-biofilm activities of Pyf A were mainly assessed against clinical CRKP isolates. Propidium iodide staining, scanning electron microscopy, glutathione (GSH) quantification, and reactive oxygen species (ROS) analysis were utilized to infer its anti-CRKP mechanism. The synergistic antibacterial effects of Pyf A and AgNO_3_ were evaluated through checkerboard and time-kill assays, as well as in vivo murine wound and catheter biofilm infection models. **Results:** Pyf A exhibited broad-spectrum antibacterial activity against ESKAPE pathogens with minimum inhibitory concentrations ranging from 0.25 to 4 μg/mL. It also showed potent anti-biofilm effects against CRKP. Pyf A disrupted the cell membranes of CRKP and markedly depleted intracellular GSH without triggering ROS accumulation. Pyf A and AgNO_3_ showed synergistic anti-CRKP activities in vitro and in vivo, by disrupting both GSH- and thioredoxin-mediated redox homeostasis. **Conclusions:** Pyf A acts as a GSH-depleting agent and, when combined with AgNO_3_, achieves dual-targeted disruption of bacterial thiol redox systems. This dual-targeting strategy enhances antibacterial efficacy of Pyf A and represents a promising therapeutic approach to combat CRKP infections.

## 1. Introduction

The advent of antibiotics has markedly increased life expectancy. However, the discovery of new antibiotics has slowed, since only 15 antibacterial agents were approved between 2000 and 2019 by the FDA [1,2]. In contrast, bacterial resistance has continued to escalate and is recognized as one of the most pressing public health threats of the 21st century. In 2019, antimicrobial resistance (AMR) was associated with approximately 5 million deaths worldwide [3]. *Klebsiella pneumoniae*, a member of the ESKAPE group of pathogens, is on the Bacterial Priority Pathogens List of the World Health Organization (WHO) [4]. According to WHO surveillance data from 2021, clinical isolates of *K. pneumoniae* were among the most frequently reported antibiotic-resistant pathogens globally [5]. In China, national surveillance data from 2005 to 2023 indicate that the rate of carbapenem-resistant *K. pneumoniae* (CRKP) has risen from 3% to approximately 26%. Among these strains, 83.6% exhibit resistance to three or more classes of antibiotics [6]. Although clinical *K. pneumoniae* isolates remain relatively susceptible to a few last-resort antibiotics, such as tigecycline and polymyxin B, resistance to these agents has been rising at an average annual rate of 1.8% over the past five years. These alarming trends underscore the urgent need for novel antibiotics or alternative antimicrobial strategies.

**Figure 1:**
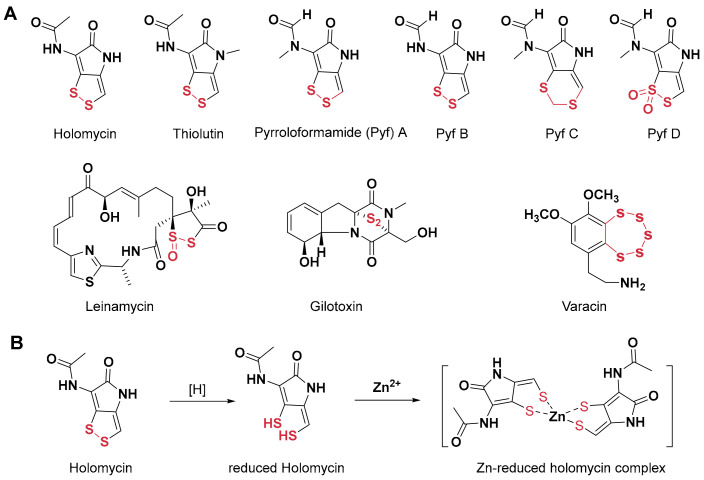
The structure and mode of actions of dithiolopyrrolones and related natural products. (**A**) Representative dithiolopyrrolones and natural products containing redox-active sulfur atoms. (**B**) The known mode of action of the prototypical holomycin: it followed reductive activation and formation of a Zn^+^ containing metallocomplex to inhibit zinc-dependent metalloproteins and disrupt intracellular metal homeostasis.

Dithiopyrrolone compounds (DTPs) represent a class of natural products characterized by a compact bicyclic scaffold, which feature a disulfide bond that confers unique electron delocalization properties (Figure 1). They also belong to a large family of natural products containing redox-active sulfur atoms, such as leinamycin, gliotoxin, and varacin [7,8,9,10,11]. Since their first discovery in the 1950s, more than 30 DTP derivatives, including holomycin and thiolutin, have been identified, which exhibit broad-spectrum antibacterial and anti-inflammatory activities [12,13]. Early studies demonstrated that DTPs strongly inhibit cellular RNA synthesis [14,15]. Recent findings further revealed that thiolutin, a representative DTP, directly inhibits RNA polymerase II [16]. Several studies have shown that the reduction of the disulfide bond to a dithiol form is essential for DTP bioactivity. Reduced DTPs can chelate various metal ions, including Zn^2+^, Mn^2+^, and Cu^2+^, thereby disrupting intracellular metal homeostasis [17,18]. Reduced DTPs typically chelate Zn^2+^ in a 2:1 stoichiometry (Figure 1), demonstrating potent inhibitory activity against zinc-dependent metalloproteins, including inhibition of the metallo-*β*-lactamase NDM-1 [17]. In eukaryotic cells, DTPs can also chelate Zn^2+^ and inhibit the deubiquitinating enzyme PSMD 14 [19], thereby stabilizing multiple intracellular proteins such as SNAIL [20].

In our previous work, we isolated and characterized four DTP natural products, pyrroloformamide (Pyf) A–D from *Streptomyces* sp. CB02980, and elucidated their biosynthetic pathway (Figure 1) [21]. Although both Pyf A and B were first identified and structurally characterized in the 1960s [22,23], Pyf C and Pyf D represent structurally novel members of the DTP family: Pyf C contains a methylene insertion within the disulfide bond, whereas Pyf D features an oxidized dithiolane in a five-membered ring, similar to a previous reported antitumor antibiotic leinamycin [8,9]. Interestingly, these unique structural modifications markedly diminish the antibacterial activity of Pyf A, suggesting the essential role of the intact disulfide bond [21].

DTPs are known to possess distinctive redox properties, with a redox potential similar to that of glutathione (GSH). In the presence of intracellular GSH, the disulfide bond in DTPs can be reduced to a dithiol form [18]. As the major endogenous thiol in bacteria, GSH plays a pivotal role in maintaining intracellular redox homeostasis. Depletion of GSH disrupts this balance, leading to the accumulation of reactive oxygen species (ROS) [24,25]. Some studies have reported that mutations in oxidative stress response-related genes are enriched in DTP-resistant yeast strains, highlighting the potential impact of DTPs on redox regulation [16]. A deeper understanding of the interactions between DTPs and bacterial redox systems may not only clarify their antimicrobial mechanisms, but also provide a theoretical foundation for developing novel antibacterial strategies targeting redox homeostasis.

In this study, we investigated the antibacterial activity of Pyf A against a range of clinical multidrug-resistant (MDR) bacteria, with a particular focus on its in vitro and in vivo efficacy against CRKP. We further explored the mechanism by examining its effects on bacterial thiol-based redox systems, including intracellular glutathione (GSH/GSSG) levels, thioredoxin reductase (TrxR) activity, and ROS production. A dual-targeting strategy against GSH and TrxR through the synergistic effects of Pyf A and AgNO_3_, a known TrxR inhibitor, were next adapted to simultaneously disrupt both GSH- and Trx-mediated redox homeostasis in CRKP. CRKP infections frequently occur in clinical settings associated with skin wounds and the use of invasive medical devices, including catheters. Therefore, we evaluated the synergistic effects of Pyf A and silver ions in a wound infection model and a catheter-associated biofilm infection model. The combination of Pyf A and silver ions demonstrated significant therapeutic efficacy against CRKP infection in mouse models. Our findings provide new mechanistic insights into the antibacterial action of Pyf A and the related DTPs, which supports their further development against CRKP infections.

## 2. Results

### 2.1. Pyf A Exhibits Potent Antimicrobial Activity Against CRKP

**Figure 2:**
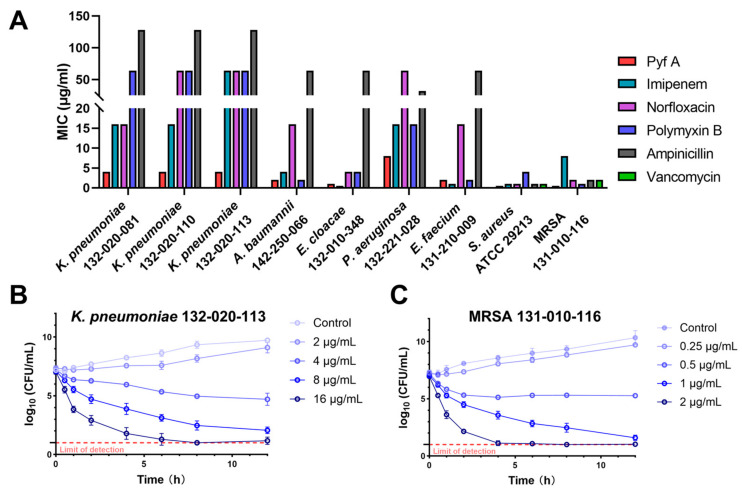
Antibacterial activity of Pyf A against clinical multidrug-resistant pathogens. (**A**) Minimum inhibitory concentrations (MICs) of Pyf A and five conventional antibiotics against ‘ESKAPE’ clinical isolates. (**B**) Time-kill curves of Pyf A against KP113 at concentrations of 2, 4, 8, and 16 μg/mL. (**C**) Time-kill curves of Pyf A against MDR SA116 at concentrations of 0.25, 0.5, 1, and 2 μg/mL.

Although several DTPs have shown promising antibacterial properties, their antimicrobial spectra vary among different analogs. The reason for these differences remains unclear, and the antibacterial potential of Pyf A has not been systematically investigated. Therefore, we first assessed the antibacterial activity of Pyf A against a panel of ESKAPE pathogens, using several clinically used antibiotics including imipenem, norfloxacin, polymyxin B, ampicillin, and vancomycin (Figure 1A, Supplementary Materials Table S1). Pyf A exhibited broad-spectrum activity against all tested clinical MDR isolates, with minimum inhibitory concentrations (MICs) ranging from 0.25 to 4 μg/mL. Time-kill assays further demonstrated that Pyf A exerted rapid bacteriostatic effects against both *K. pneumoniae* 132-020-113 (KP113) and *Staphylococcus aureus* 131-010-116 (Figure 2B,C). Pyf A exhibited notably lower MICs against several multidrug-resistant strains compared to conventional antibiotics.

Interestingly, Pyf A showed consistent and potent activity against multiple clinical MDR *K. pneumoniae* isolates, all with a MIC of 4 μg/mL (Supplementary Materials Table S1). Resistance profiling revealed that these strains were highly resistant to multiple antibiotic classes. KP113 even showed resistance to imipenem (Supplementary Materials Figure S1 and Table S2). Given the urgent clinical threat posed by CRKP, KP113 was selected as the representative strain for subsequent mechanistic and therapeutic investigations. This selection would offer a clinically relevant model that mirrors the resistance profile of high-risk CRKP strains, thereby enabling a more accurate evaluation of the therapeutic potential of Pyf A.

### 2.2. Pyf A Inhibits Biofilm Formation and Disrupts Established Biofilms

**Figure 3:**
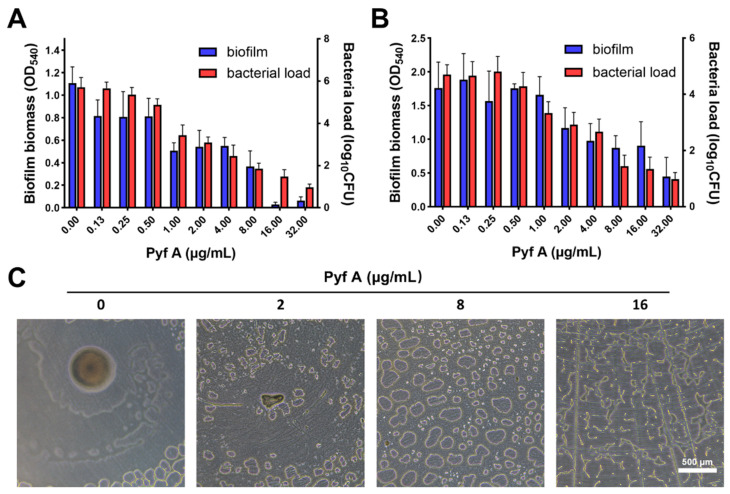
Effects of Pyf A on biofilm formation in KP113. (**A**) Inhibition of biofilm formation by Pyf A at various concentrations. Biofilm biomass was quantified by crystal violet staining (OD_540_), and bacterial load in the supernatant was measured by CFU counting. (**B**) Disruption of pre-formed mature biofilms by Pyf A. After 48 h biofilm formation, biofilms were treated with Pyf A for 12 h, followed by biomass and bacterial load quantification as in (**A**) (*n* = 6). (**C**) Bright-field microscopy images showing structural changes in biofilms following treatment with increasing concentrations of Pyf A (0, 2, 8, and 16 μg/mL). Scale bar: 500 μm. Data in (**A**) and (**B**) represent mean ± SD (*n* = 6).

We evaluated the biofilm inhibition activity of Pyf A using crystal violet staining to quantify total biofilm biomass, and colony-forming unit (CFU) enumeration to assess bacterial load within the biofilm. Pyf A markedly inhibited KP113 biofilm formation at sub-MIC concentrations, while almost complete suppression was observed at 2 × MIC (Figure 3A). Notably, Pyf A also demonstrated potent activity against mature biofilms. At its MIC concentration, Pyf A significantly reduced both biofilm biomass and bacterial load within the biofilm (Figure 3B). Microscopic observation further confirmed the disruption of the biofilm structure by Pyf A treatment, indicating its strong anti-biofilm efficacy (Figure 3C).

### 2.3. Pyf A Compromises Bacterial Membrane Integrity

**Figure 4:**
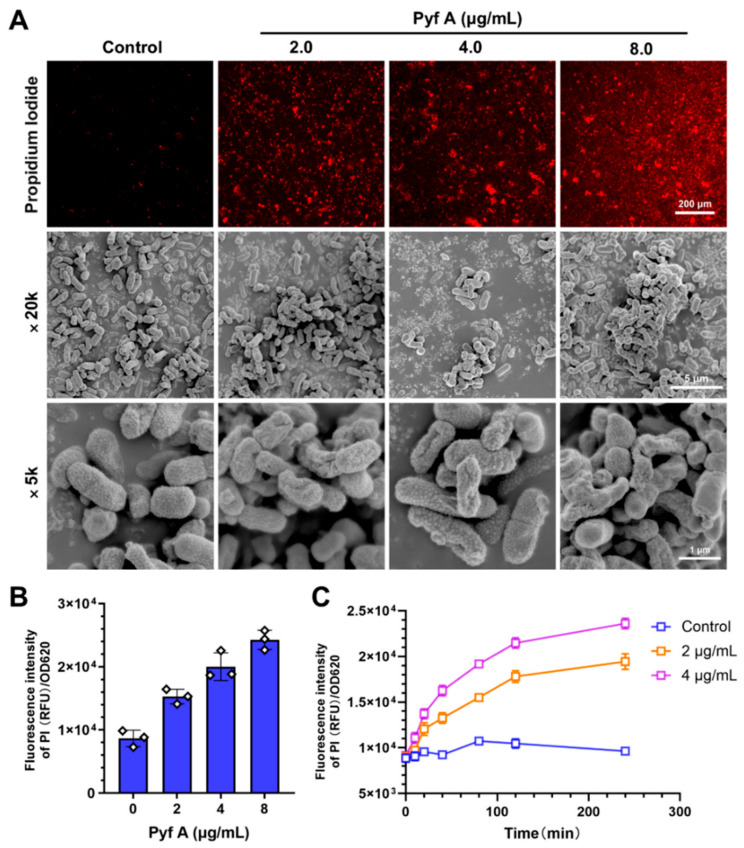
Pyf A disrupts the membrane integrity of *K. pneumoniae* in a dose- and time-dependent manner. (**A**) KP113 was treated with increasing concentrations of Pyf A (2, 4, and 8 μg/mL) for 4 h. Membrane damage was assessed by propidium iodide (PI) staining and scanning electron microscopy (SEM). (**B**) Quantification of PI fluorescence intensity after 4 h treatment with Pyf A, normalized to OD_600_. Data represent mean ± SD (*n* = 3), dots represent individual data points. (**C**) Time-course of PI fluorescence intensity in *K. pneumoniae* treated with Pyf A at 2 and 4 μg/mL.

Since bacterial membrane is the primary barrier for maintaining cellular homeostasis, the disruption of its permeability often results in leakage of cytoplasmic contents, metabolic dysfunction, and ultimately cell death. Furthermore, membrane-targeting antimicrobial agents are considered less susceptible to resistance development, representing a promising strategy for novel antibiotic discovery [26]. To investigate whether membrane disruption contributes to the antibacterial mechanism of Pyf A, we assessed its effects on the integrity of *K. pneumoniae* membrane using propidium iodide (PI) staining and scanning electron microscopy (SEM). The short-term (4 h) exposure of KP113 to Pyf A induced a dose-dependent increase in PI fluorescence intensity (Figure 4A,B), indicative of compromised membrane permeability. A time-dependent increase in fluorescence was also observed, with the fluorescence intensity peaking approximately 4 h after treatment (Figure 4C). Consistently, SEM imaging revealed morphological abnormalities in bacterial cells following Pyf A exposure, including cellular collapse, membrane rupture, and surface shrinkage (Figure 4A). Together, these observations suggest that Pyf A disrupts membrane integrity, which likely contributes to its bactericidal activity.

### 2.4. Pyf A Reduces Intracellular GSH Without Elevating ROS

**Figure 5:**
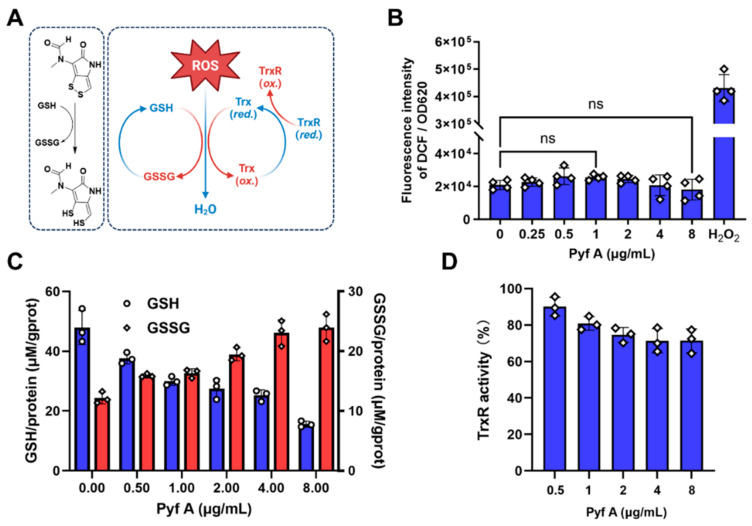
Pyf A depletes glutathione without inducing ROS accumulation and exhibits partial TrxR inhibitory activity. (**A**) Schematic of the cellular antioxidant systems and their interplay in scavenging ROS. Pyf A is proposed to disrupt the glutathione (GSH/GSSG) systems, potentially leading to oxidative imbalance. (**B**) Measurement of intracellular ROS levels in KP113 after treatment with Pyf A. H_2_O_2_ (1 mM) was used as a positive control (*n* = 4). (**C**) Quantification of intracellular GSH and GSSG levels in KP113 treated with Pyf A. blue bars represent GSH levels, and red bars represent GSSG levels. Pyf A induced a dose-dependent decrease in GSH and increase in GSSG. (**D**) Pyf A modestly inhibit TrxR enzymatic activity (*n* = 3). Data represent mean ± SD; ns, not significant.

DTPs require intracellular reduction by GSH to generate their activated dithiols, which may thereby deplete cellular GSH (Figure 5A) [16]. This implies that DTP would interfere with intracellular redox homeostasis. To examine the redox-related effects of Pyf A in *K. pneumoniae*, we assessed both intracellular ROS levels and GSH/GSSG ratios in KP113 following Pyf A treatment. Treatment with Pyf A across a concentration range from 0.125 × MIC to 2 × MIC (0.25–8 μg/mL) did not significantly increase intracellular ROS levels. In contrast, we observed a marked depletion of total GSH and a decrease in the GSH/GSSG ratio (Figure 5C), indicating significant alteration of the redox balance. These results suggest that Pyf A exerts a pronounced disruption of the redox environment in *K. pneumoniae,* despite stable ROS levels.

Typically, bacterial redox homeostasis is regulated by two major antioxidant systems: the glutathione system and the thioredoxin system. These systems function in a partially redundant manner to sustain a reducing intracellular environment, mitigate exogenous oxidative stress, and preserve protein functions (Figure 5A) [27]. We next assessed whether Pyf A inhibits thioredoxin reductase (TrxR) activity. Biochemical assays revealed only modest inhibition: TrxR retained 71.4 ± 5.3% of its enzymatic activity at 2 × MIC of Pyf A (8 μg/mL) (Figure 5D). As a key component of the thioredoxin system in bacteria, TrxR also directly detoxifies ROS and regenerates reduced thioredoxin (Trx) to sustain the intracellular reducing state (Figure 5A) [24]. These findings indicate that Pyf A selectively depletes intracellular GSH without triggering a corresponding increase of ROS, likely attributable to compensation by the thioredoxin system.

### 2.5. Pyf A and AgNO_3_ Synergistically Induce ROS and Bactericidal Activity

**Figure 6:**
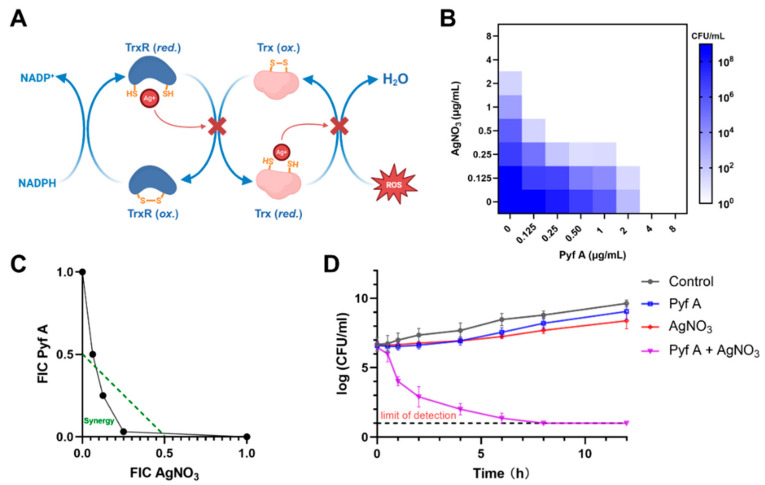
Synergistic bactericidal activity of Pyf A and AgNO_3_ through disruption of redox homeostasis and induction of ROS. (**A**) Proposed mechanism of ROS generation and redox system disruption by Pyf A and AgNO_3_. Pyf A perturbs the glutathione (GSH/GSSG) cycle, while AgNO_3_ inhibits the thioredoxin (Trx/TrxR) system. Their combined action leads to oxidative stress and ROS accumulation. (**B**) Heat map showing the bacterial viability (CFU/mL) under different concentrations of Pyf A and AgNO_3_. Darker shades indicate higher CFU counts. (**C**) Isobologram showing the fractional inhibitory concentration (FIC) indices of Pyf A and AgNO_3_. Data points below the green line indicate synergistic interaction (FIC index < 0.5). (**D**) Time-kill curves of KP113 treated with Pyf A, AgNO_3_, or their combination. The combination led to a rapid and sustained decrease in bacterial load below the detection limit. Data represent mean ± SD (*n* = 3).

The bacterial thioredoxin system comprises TrxR (also known as TrxB) and two thioredoxins (TrxA and TrxC). TrxR employs NADPH to reduce TrxA/TrxC via its disulfide redox-active site, driving essential reductive processes in DNA synthesis and protein repair [27]. Silver ions have been reported as potent inhibitor of the thioredoxin system (Figure 6A). In vitro enzymatic assays demonstrated that silver ions inhibit TrxR and Trx with dissociation constants of 1.4 μM and 1.5 μM, respectively (~0.238 μg/mL and ~0.255 μg/mL of AgNO_3_) [28]. We observed that AgNO_3_ exhibits potent inhibitory activity against KP113 with an IC_50_ value of approximately 0.5 μg/mL, probably through its interaction with TrxR (Figure 6C). Therefore, we envision that dual inhibition of both glutathione and thioredoxin systems in *K. pneumoniae* may have synergistic antibacterial effects.

We first evaluated the antibacterial activity of Pyf A and AgNO_3_ against KP113 using a checkerboard dilution assay (Figure 6B,C). In the presence of 0.5 μg/mL AgNO_3_, the MIC of Pyf A decreased from 4 to 0.125 μg/mL. Conversely, co-treatment with Pyf A at 0.5 μg/mL significantly decreased the MIC of AgNO_3_ from 2 to 0.5 μg/mL. Consistent with these findings, time-kill assays further demonstrated a pronounced synergistic bactericidal effect between the two agents (Pyf A 0.5 μg/mL, AgNO_3_ 0.5 μg/mL) (Figure 6D).

**Figure 7:**
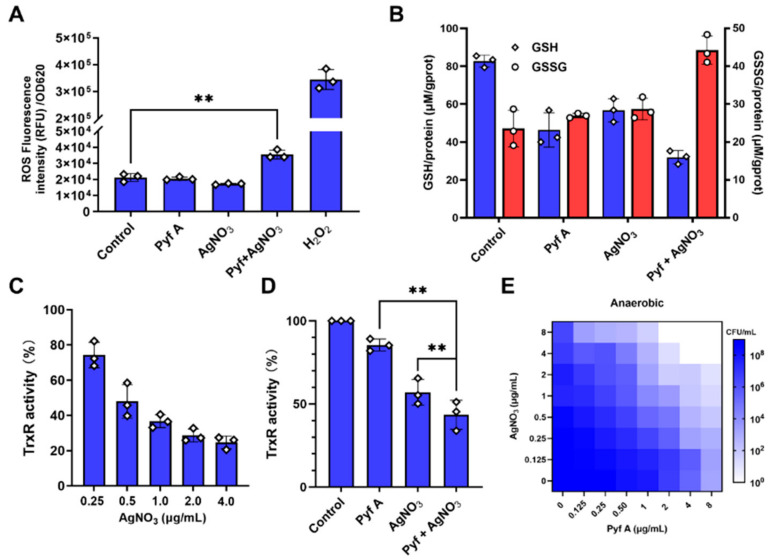
Combination of Pyf A and AgNO_3_ induces ROS accumulation, redox imbalance, and enhanced antibacterial activity. (**A**) Intracellular ROS levels measured in KP113 treated with Pyf A (0.5 μg/mL), AgNO_3_ (0.5 μg/mL), or their combination. H_2_O_2_ (1 mM) served as a positive control. (**B**) Intracellular levels of reduced (GSH) and oxidized (GSSG) glutathione upon indicated treatments. blue bars represent GSH levels, and red represent GSSG. (**C**) Dose-dependent inhibition of TrxR activity by AgNO_3_. (**D**) TrxR activity in bacterial lysates treated with Pyf A (0.5 μg/mL), AgNO_3_ (0.5 μg/mL), or both. (**E**) Heatmap showing bacterial viability (CFU/mL) after treatment with various combinations of Pyf A and AgNO_3_. Lighter shades indicate greater antibacterial activity. Data represent mean ± SD (*n* = 3); *p* < 0.01 (**).

We further investigated the effects of the drug combination on bacterial redox homeostasis. At sub-MIC concentrations (0.5 μg/mL for each compound), the combination of Pyf A and AgNO_3_ notably induced intracellular ROS accumulation (Figure 7A). Compared to either agent alone, the combination resulted in greater depletion of intracellular glutathione levels and significantly inhibited TrxR activity (Figure 7B,C). To determine whether the synergistic effect was oxygen-dependent, we evaluated the antibacterial activity of Pyf A and AgNO_3_ under anaerobic conditions. As a result, the synergistic antibacterial effect was accordingly diminished. These data suggest that sustained ROS accumulation is a major contributor to the bactericidal synergy for Pyf A and AgNO_3_. By concurrently targeting the glutathione and thioredoxin systems in *K. pneumoniae*, the combination compromises bacterial antioxidant defenses, thus leading to oxidative damage and potent antibacterial activity.

**Figure 8:**
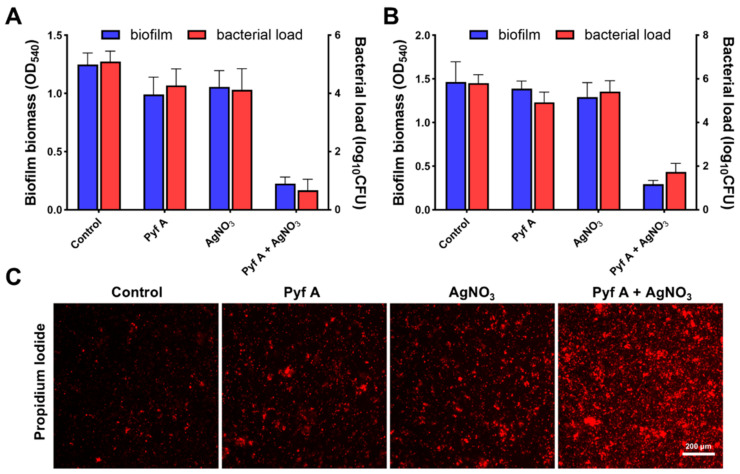
The anti-biofilm effects of Pyf A and AgNO_3_ against KP113. (**A**) Inhibition of biofilm formation by Pyf A (0.5 μg/mL), AgNO_3_ (0.5 μg/mL), or their combination. Biofilm biomass was quantified by crystal violet staining (OD_450_), and bacterial load in the supernatant was measured by CFU counting (*n* = 6). (**B**) Disruption of pre-formed mature biofilms by Pyf A (0.5 μg/mL), AgNO_3_ (0.5 μg/mL), or their combination. After 48 h biofilm formation, biofilms were treated for 12 h, followed by biomass and bacterial load quantification as in (**A**) (*n* = 6). (**C**) Representative fluorescence microscopy images of PI-stained biofilms. Red fluorescence denotes dead or membrane-damaged cells. Scale bar = 200 μm.

This drug combination also effectively inhibited biofilm formation and disrupted established biofilms of KP113 (Figure 8A and Figure S2). Live/dead bacterial staining observed via laser scanning confocal microscopy further confirmed rapid bacterial killing within mature biofilms (Figure S3). In addition, PI staining indicated that the treatment significantly compromised bacterial membrane integrity (Figure 8C and Figure S4).

### 2.6. The Combination of Pyf A and AgNO_3_ Is Effective in Mouse Models

**Figure 9:**
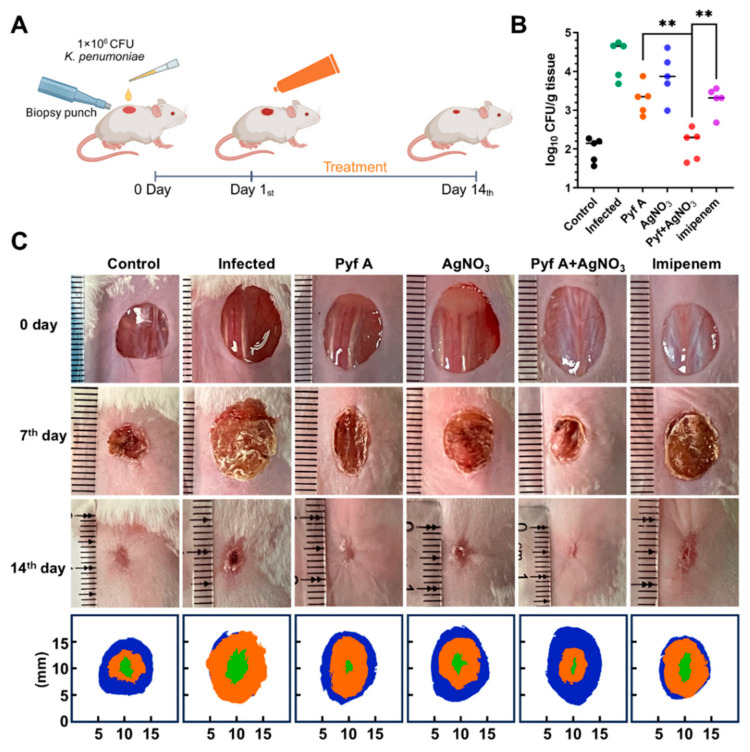
Pyf A combined with AgNO_3_ promotes wound healing and reduces bacterial burden in a murine skin infection model by *K. pneumoniae*. (**A**) The schematic form of the wound infection model. Animals were subjected to full-thickness excisional wounds and inoculated with KP113 (1 × 10^6^ CFU/wound, 24 h), followed by topical treatment from day 1 to day 14. Pyf A ointment (0.5 mg/mL), AgNO_3_ (0.5 mg/mL), combination therapy (Pyf A 0.5 mg/mL + AgNO_3_ 0.5 mg/mL), and imipenem (1 mg/mL). The vehicle control and infected group received blank ointment base. (**B**) Bacterial burden in infected tissues (log CFU/g) after 7 days of treatment. The combination significantly reduced bacterial load, compared to single-drug and imipenem (*n* = 5). (**C**) Representative images of wounds at day 0, 7, and 14 post-treatment, alongside corresponding wound size maps. The combination group showed markedly improved healing and smaller wound areas (*n* = 3). Data represent mean ± SD; *p* < 0.01 (**).

To evaluate the in vivo antibacterial and anti-biofilm effects of Pyf A and AgNO_3_ combination, we established mouse models of skin wound infection and subcutaneous catheter biofilm infection using KP113. In the skin wound infection model, wounds in untreated group exhibited typical infection symptoms and delayed healing during the observation period from days 0 to 7 as compared to uninfected group. In contrast, both single (Pyf A or AgNO_3_) and the combination treatments markedly alleviated infection and promoted faster wound healing (Figure 9C). On day 7, the combination treatment group showed lower bacterial loads compared to the single treatment groups and imipenem group (Figure 9B). Additionally, the combination treatment group exhibited the highest wound healing rate. Fewer inflammatory cells were detected in histological evaluation, revealing reduced inflammation in the combination group and Pyf A group. Masson staining further confirmed that the combination treatment enhanced the deposition of regular and dense collagen fibers by promoting tissue regeneration (Figure S5).

**Figure 10:**
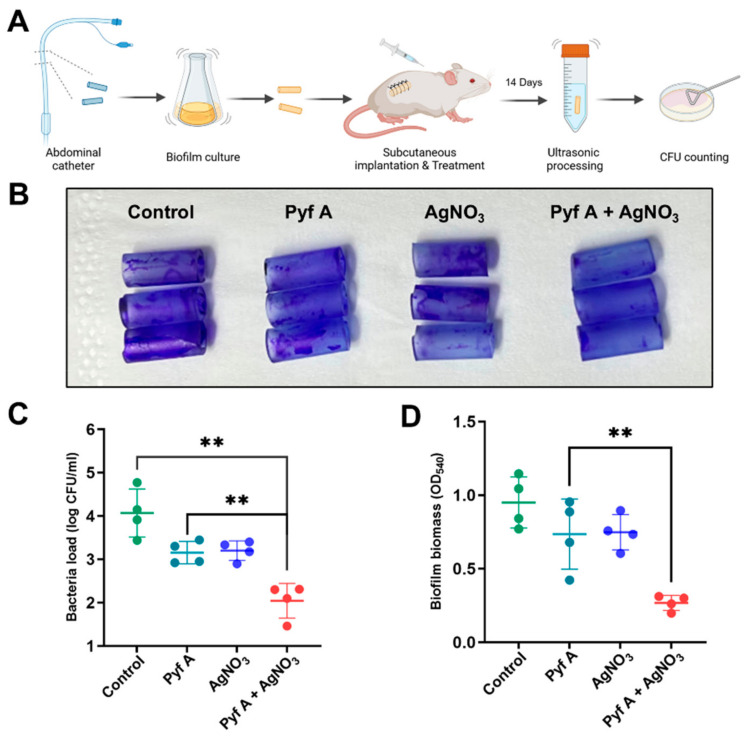
Combination of Pyf A and AgNO_3_ effectively eradicates biofilm-associated catheter infection model of *K. pneumoniae*. (**A**) Schematic of catheter-associate biofilm model. The treatment groups included the following: Pyf A (0.5 mg/mL), AgNO_3_ (0.5 mg/mL), combination therapy (Pyf A 0.5 mg/mL + AgNO_3_ 0.5 mg/mL), and vehicle control (saline). (**B**) Representative crystal violet-stained images of catheters collected at the endpoint of the experiment. (**C**) Bacterial load (log_10_ CFU/mL) on catheters after 14 days. (**D**) Biofilm biomass quantification: crystal violet-stained catheters from each animal were washed, destained with ethanol, and measured at OD_540_. Data represent mean ± SD (*n* = 4), *p* < 0.01 (**).

In the subcutaneous catheter biofilm infection model, treatments were administered 24 h post-infection via subcutaneous injection (200 μL per dose) adjacent to the implanted catheter at day 0, 3, and 7. Catheters were collected and examined at the end of the experiment, and the surrounding tissue was assessed for infection. The untreated group exhibited severe infection, fluid accumulation, and adhesion, whereas the combination treatment significantly alleviated these symptoms (Figure S5). Furthermore, biofilm adherence was markedly lowered in the combination group, compared to the untreated group (Figure S6). The bacteria load also demonstrated a potent antibacterial and antibiofilm efficacy in vivo of the drug combination (Figure 10C,D).

## 3. Discussion

Despite the discovery of Pyf A in 1969, its antibacterial activity and mechanism have not been systematically investigated. In this study, we discovered that Pyf A exhibited superior in vitro activity against ESKAPE pathogens to several “last-resort” antibiotics, including polymyxin B and vancomycin. Mechanically, Pyf A depleted intracellular GSH without triggering ROS accumulation in *K. pneumoniae*. PyfA and AgNO_3_ further exhibited synergistic antibacterial effects against KP113, a clinical CRKP isolate, by concurrently targeting the GSH/GSSG and TrxR systems. The drug combination also demonstrated remarkable efficacy in KP113-infected murine skin and catheter-associated biofilm models. These data not only revealed the antibacterial mechanism of Pyf A, but also revealed a promising drug combination strategy of this half-century-old antibiotic and silver ion for the treatment of CRKP infection, one of the deadliest bacterial pathogens lacking effective drug treatment.

Sulfur plays a crucial role in redox-related biological processes due to its variable oxidation states [7,29]. Small molecule thiol redox systems are widely conserved in both prokaryotic and eukaryotic cells. These systems play an essential role in maintaining redox homeostasis during various biological processes [30,31]. Disulfide bonds are also commonly present in natural products, such as leinamycin and epidithiodiketopiperazine alkaloids. They are known for their marked biological activity, mainly attributed to the redox properties of their disulfide bonds [32,33]. DTPs typically possess a structurally conserved pyrrolidone-disulfide heterocyclic pentadiene scaffold, in which the disulfide bond also functions as the pharmacophore [12]. Previous studies have mainly focused on the reduction of disulfide bond to thiol groups and their effects on cellular metal ion homeostasis and protein functions [17,19]. However, the impact of this reduction on bacterial redox homeostasis has yet to be fully characterized.

Herein, we discovered that Pyf A could deplete intracellular GSH, while the treated *K. pneumoniae* did not accumulate ROS (Figure 5). This result aligned with two recent studies that holomycin has a lower redox potential than GSH, which can be reduced in the presence of cellular thiols or reductases [18,34,35,36]. Since the glutathione and thioredoxin redox systems interact and complement each other in most prokaryotic and eukaryotic organisms [27,31], the thioredoxin system in *K. pneumoniae* may still retain ROS scavenging capacity to keep a normal cellular ROS level. In addition, Pyf A only had limited inhibitory activity (71.42% ± 5.13) at 2 × MIC against bacterial thioredoxin reductase (Figure 5D). Furthermore, a mutagenomic approach has revealed notable alterations in oxidative stress-related genes in holomycin-resistant yeast strains, including thioredoxin reductase 1 (*trr1*). Knocking out the *trr1* gene led to increased susceptibility to holomycin [16], further indicating that the thioredoxin redox pathway may play a critical role in bacterial sensitivity to DTPs.

Silver ions are known inhibitors of the thioredoxin system (*K_d_* = 0.238 μg/mL), and they significantly reduce the reductase activity and oligomerization of TrxR [27,37]. We therefore explored a drug combination strategy of Pyf A and silver ions to concurrently disrupt the glutathione and thioredoxin systems (Figure 6, Figure 7 and Figure 8). Checkboard assays and FIC index analysis support a synergistic interaction between Pyf A and silver ions. Time-kill studies further revealed that the combination of Pyf A and AgNO_3_ (both 0.5 μg/mL) rapidly killed KP113 and caused substantial membrane damage. Furthermore, the combination effectively inhibited the biofilm formation of KP113 and eradicated the mature biofilms. Mechanistically, this combination significantly reduced the GSH/GSSG ratio and suppressed TrxR activity, confirming the concurrent disruption of two major redox systems in *K. pneumoniae* and a consequent increase of intracellular ROS. Notably, the synergistic effect was nearly abolished under anaerobic conditions, highlighting the essential role of oxygen and ROS in mediating this synergistic antibacterial effect.

Wound infections caused by *K. pneumoniae* are frequently encountered in clinical practice, particularly in postoperative wounds, burn injuries, or chronic ulcers. *K. pneumoniae* also plays a major role in device-associated infections due to its capacity to form persistent biofilms on medical devices [38]. Within biofilms, bacteria adopt a dormant metabolic state and exhibit marked drug resistance, diminishing the efficacy of conventional antibiotics by 10- to 1000-fold [39]. The emergence and spread of CRKP further compound this challenge [40,41]. Therefore, we employed both murine skin wound and subcutaneous catheter-associated biofilm infection models to evaluate if the Pyf A–AgNO_3_ combination exhibits potent in vivo anti-CRKP and anti-biofilm effects (Figure 9 and Figure 10). Female mice were selected to avoid potential aggressive behaviors among group-housed male mice, especially in wound infection models where skin integrity is involved. The treatment significantly alleviated CRKP infection symptoms, accelerated wound healing and tissue recovery, and effectively eradicated biofilms on implanted catheters. Notably, in the catheter model, both Pyf A alone and the combination markedly reduced catheter adhesion and local inflammation compared to the untreated group (Figures S5 and S6). This may be partially attributed to the potential anti-inflammatory properties of Pyf A. Previous studies have reported that the inhibition of deubiquitinating enzyme PSMD14 (Rpn11) can suppress NLRP3 inflammasome activation, suggesting a possible immunomodulatory advantage of Pyf A that merits further investigation.

In summary, our study demonstrates that Pyf A is a promising antibacterial agent with potent in vitro and in vivo efficacy against CRKP. Pyf A functions as a glutathione depleting agent, while it could synergistically disrupt both the GSH and thioredoxin antioxidant systems when combined with the TrxR inhibitor AgNO_3_. This dual-targeting strategy leads to a significant elevation of intracellular ROS levels in treated CRKP in vitro and a corresponding enhancement of bactericidal activity with reduced dosages. Therefore, the dual redox-targeting strategy represents a promising avenue for the further development of Pyf A and other DTPs in combating CRKP and other drug-resistant ESKAPE pathogens.

## 4. Materials and Methods

### 4.1. Bacterial Strains and Culture Conditions

All the clinical isolates of *Klebsiella pneumoniae*, *Staphylococcus aureus*, *Escherichia coli*, *Enterococcus faecium*, *Acinetobacter baumannii*, and *Pseudomonas aeruginosa* were obtained from Xiangya Hospital, Changsha, China. These strains were cultured in Mueller–Hinton (MH) broth or agar. All bacterial cultures were incubated at 37 °C with shaking at 220 rpm or static culture. The bacterial concentration was determined using the McFarland turbidity standard [42] and confirmed by plate counting. For each experiment, the bacteria were grown to the appropriate phase of growth before use.

### 4.2. Chemicals and Reagents

Pyrroloformamide A/B (Pyf A/B) were fermented, isolated, and purified from the *Streptomyces* sp. CB02980, with approximately yields of 32.6 and ~10 mg/L and a final purity of ≥95% by HPLC, respectively [21]. Silver nitrate (purity 99.5%) was purchased from Sinopharm, Co., Ltd. (Beijing, China). The following antibiotics were used as controls: imipenem, norfloxacin, ampicillin, vancomycin, and polymyxin B, all of which were obtained from Energy Chemical Co., Ltd. (Shanghai, China).

### 4.3. Antimicrobial Susceptibility Testing and MIC Determination

Antimicrobial susceptibility was assessed using the disk diffusion method and broth dilution method, following the guidelines outlined by the European Committee on Antimicrobial Susceptibility Testing (EUCAST). The minimum inhibitory concentration (MIC) of Pyf A and other antibiotics was determined by the broth microdilution method. Briefly, serial two-fold dilutions of the compounds were prepared in MH broth, and bacterial suspensions were added to each well. The MIC was defined as the lowest concentration of the compound that inhibited bacterial growth after incubation at 37 °C for 18–24 h, and resazurin (Yuanye Bio-Technology Co., Ltd., Shanghai China) staining was performed to visually assess bacterial growth inhibition, with deep blue color indicating an absence of bacteria growth [43,44].

### 4.4. Time-Kill Assay

*K. pneumoniae* cultures were grown to the logarithmic phase and then diluted in MH broth to a McFarland turbidity of 0.2 (approximately 1 × 10^6^ CFU/mL). The *K. pneumoniae* suspensions were subsequently treated with the respective drugs in each experimental group, with each group containing three replicates. At specified time points (0.5, 1, 2, 4, 6, 8, and 12 h), aliquots were taken, serially diluted, and plated on M.H. agar to determine CFU. The bactericidal activity was assessed by evaluating the reduction in CFU compared to untreated controls [45,46].

### 4.5. Biofilm Inhibition and Pre-Formed Biofilm Clearance Assay

*K. pneumoniae* biofilm assays were performed using a 96-well microtiter plate model. *K. pneumoniae* cultures were grown to the logarithmic phase and diluted in M.H. broth to a McFarland standard of 0.5 (approximately 1 × 10^8^ CFU/mL). For the biofilm inhibition assay, 200 μL of the *K. pneumoniae* suspension was added to each well, followed by the addition of test compounds. The plates were incubated at 37 °C with shaking at 180 rpm for 48 h. After incubation, the biofilm was gently washed with saline to remove planktonic cells, and then stained with 0.1% crystal violet solution for 15 min. After washing to remove excess stain, the bound crystal violet was solubilized with 100 μL of ethanol per well, and the absorbance was measured at 450 nm to quantify biofilm biomass. *K. pneumoniae* loads were quantified via CFU counts after biofilm dissociation (100 μL saline, ultrasonication, and pipette vortexing) in parallel experiments. For the pre-formed biofilm clearance assay, *K. pneumoniae* suspensions were incubated for 48 h to allow mature biofilm formation. Subsequently, the wells were gently washed to remove non-adherent cells, and fresh medium containing test compounds was added. After 12 h of treatment, biofilm biomass and *K. pneumoniae* load were assessed as described above. All experiments were conducted with 8 replicates per group (*n* = 8) [47,48,49].

### 4.6. Confocal Laser Scanning Microscopy

*K. pneumoniae* biofilm viability and structural morphology were examined using confocal laser scanning microscopy. Mature biofilms were cultivated in glass-bottom culture dishes. After 48 h of incubation, the biofilms were gently washed with saline and treated with the indicated compounds for approximately 4 h at 37 °C. Following treatment, the biofilms were stained using the Live/Dead Viability Kit (cat: PF0007, Proteintech Group, Wuhan, China), according to the manufacturer’s protocol. Fluorescent images of the stained biofilms were captured using a confocal laser scanning microscope. Representative fields were selected for qualitative assessment. Three-dimensioinal reconstructions of representative biofilm images were performed using Image-Pro Plus software version 6.0 (Media Cybernetics, Rockville, MD, USA) [48,50].

### 4.7. Scanning Electron Microscopy

*K. pneumoniae* suspension was diluted to approximately 1 × 10^8^ CFU/mL in fresh medium. *K. pneumoniae* were then treated with the indicated compounds for 12 h at 37 °C. Following treatment, bacteria were harvested by centrifugation at 5000× *g* for 10 min and washed twice with saline. The collected bacteria were fixed with 2.5% glutaraldehyde at 4 °C overnight, followed by a graded ethanol dehydration series (30%, 50%, 70%, 90%, and 100%, 15 min each step). After complete dehydration, the samples were dried, sputter-coated with a thin layer of gold, and examined using a scanning electron microscope operated at an accelerating voltage of 10 kV under high-vacuum conditions. Representative images were captured at magnifications ranging from 5000× to 20,000× [51].

### 4.8. PI Uptake Assay

*K. pneumoniae* cultures were diluted to approximately 1 × 10^6^ CFU/mL in fresh MH broth. A total volume of 2 mL per group was treated with the indicated compounds for 4 h at 37 °C with shaking (220 rpm). After treatment, the cells were harvested by centrifugation and washed twice with PBS, followed by staining with PI. Stained cells were transferred to black, flat-bottom 96-well plates (200 μL per well), and fluorescence intensity was measured using a microplate reader (*Ex*: 535 nm, *Em*: 617 nm). The relative fluorescence was normalized to bacteria density determined by OD_620_. In parallel, PI-stained samples were observed under a fluorescence microscope to visualize cell membrane damage [52,53].

### 4.9. ROS Measurement

Intracellular ROS levels in *K. pneumoniae* were measured using a ROS detection kit (Cat. S0033S, Beyotime Biotechnology, Shanghai, China) according to the manufacturer’s instructions. Briefly, *K. pneumoniae* cultures were diluted to approximately 1 × 10^6^ CFU/mL in MH broth. After treatment with the indicated compounds for 4 h at 37 °C with shaking (220 rpm), bacterial cells were harvested by centrifugation and washed twice with saline. The cells were then incubated with the ROS-sensitive fluorescent probe provided in the kit for 30 min at 37 °C in the dark. After incubation, fluorescence intensity was measured using a microplate reader (*Ex*: 488 nm, *Em*: 525 nm). The relative fluorescence was normalized to cell density determined by OD_620_. A quantity of 1 mM H_2_O_2_, 20 min treatment was used as a positive control for ROS induction. Each experimental condition was tested in 4 replicates (*n* = 4) [54].

### 4.10. GSH/GSSG Quantification

Quantification of intracellular reduced glutathione and oxidized glutathione levels in *K. pneumoniae* was performed using a commercial GSH/GSSG assay kit (Cat. S0053, Beyotime Biotechnology, China) according to the manufacturer’s protocol. *K. pneumoniae* cultures were grown and treated. After treatment, cells were harvested by centrifugation, washed twice with saline, and lysed using the protein removal reagent provided in the kit. Total glutathione (GSH + GSSG) and GSSG were measured via a DTNB (5,5′-dithiobis-(2-nitrobenzoic acid)) recycling method following kit instructions. The GSH concentration was calculated by subtracting GSSG from total glutathione. Absorbance was measured at 412 nm using a microplate reader. Each experimental condition was performed in 4 biological replicates (*n* = 4) [55,56].

### 4.11. TrxR Activity Assay

TrxR activity was measured using a colorimetric TrxR assay kit (Cat. No. KTB1650, Abbkine, Wuhan, China) following the manufacturer’s instructions. Briefly, *K. pneumoniae* cultures were grown and treated as described in previous sections. After treatment, cells were harvested by centrifugation, washed with ice-cold saline, and lysed in the assay buffer provided with the kit. The assay is based on the TrxR-catalyzed reduction of DTNB to TNB, which produces a yellow color measurable at 412 nm. Absorbance was recorded using a microplate reader, and TrxR activity was calculated according to the kit’s protocol. Each condition was tested in 4 biological replicates (*n* = 4) [56].

### 4.12. Drug Combination and Checkboard Assay

For the drug combination assays, *K. pneumoniae* cultures were grown to the logarithmic phase and diluted to approximately 1 × 10^6^ CFU/mL. The diluted bacterial suspensions were then distributed into a 96-well microplate (200 μL per well) in an 8 × 8 format. Various concentrations of the drug combinations were added to each well. The plate was incubated at 37 °C for 24 h. After incubation, 100 μL of bacterial suspension from each well was collected, serially diluted, and plated on agar plates for CFU counting. In parallel, a portion of the *K. pneumoniae* samples was stained with resazurin to assess *K. pneumoniae* growth inhibition. The fractional inhibitory concentration index (*FIC*_index_) was calculated to evaluate the synergistic effect of the drug combinations using the following formula:

$${FIC}_{\mathrm{index}}=\left({FIC}_{\mathrm{PyfA}}+{FIC}_{{\mathrm{Ag}}^{+}}\right)=\left(\frac{{MIC}_{\mathrm{combination},\mathrm{PyfA}}}{{MIC}_{\mathrm{PyfA}}}\right)+\left(\frac{{MIC}_{\mathrm{combination},\mathrm{AgNO}3}}{{MIC}_{\mathrm{AgNO}3}}\right)$$

*FIC*_index_ ≤ 0.5 indicates synergy, *FIC*_index_ > 0.5 but < 1 indicates additive effects, and *FIC* index > 1 suggests antagonism [45,57].

### 4.13. In Vivo Models

All animal experiments were conducted using female ICR mice (4–6 weeks old), obtained from Hunan Slack Jingda Laboratory Animal Co., Ltd. (Changsha, China). The animals were housed under specific pathogen-free (SPF) conditions at the Experimental Animal Center of Central South University with controlled temperature (25 ± 2 °C), relative humidity (50 ± 10%), and a 12-h light/dark cycle. Sterilized feed and acidified water were provided ad libitum. All experimental procedures were approved by the Institutional Animal Care and Use Committee (IACUC) (Approval No. 2020SYDW169, No. 2023SYDW0274).

Murine Skin Infection Model: For the excisional wound infection model, a full-thickness skin wound (diameter 1 cm) was created on the dorsal surface using biopsy punch. The wounds were inoculated with 1 × 10^6^ CFU of KP113 in 100 μL of saline. After 24 h of bacterial inoculation, mice were randomly divided into groups (*n* = 8), and the treatment was administered every two days. On day 7, 5 mice from each group were sacrificed, and the wound tissues were divested, weighed, and homogenized in saline. The resulting supernatant was serially diluted and plated to determine bacterial CFU per gram of tissue. The remaining mice were monitored on days 0, 7, and 14 for wound healing progression, with photographs taken at each time point. Wound area was analyzed using ImageJ version 1.52a, (NIH, Bethesda, MD, USA), and wound closure rates were calculated to generate representative wound healing merge picture. Histological evaluations, including H&E and Masson staining, were performed on divested skin samples [58,59].

Catheter Biofilm Infection Model: KP113 were cultured to logarithmic phase. Then sterile silicone catheters (Huatai Medical Equipment Co., Ltd., Taizhou, China) were cut into 1 cm segments and incubated in the bacterial suspension for 48 h under shanking conditions to allow biofilm formation. After gentle washing, biofilm-coated catheter segments were subcutaneously implanted into dorsal region of the mice. After 24 h infection, mice were divided into treatment groups (*n* = 8), and treated via subcutaneous injection on days 0 and 3. On day 7, mice were sacrificed, and catheters were collected. For half the mice (*n* = 4), catheters were washed and subjected to crystal violet staining, followed by ethanol solubilization and OD_450_ measurement to quantify residual biofilm biomass. The remaining catheters (*n* = 4) were washed, sonicated in sterile saline to dislodge adherent bacteria, and the suspensions were plated for bacterial CFU counting [60,61].

### 4.14. Statistical Analysis

All statistical analyses were performed using GraphPad Prism 10.0 (GraphPad Software, San Diego, CA, USA). Data are expressed as mean ± standard deviation (SD) from at least three independent biological replicates. For comparisons between two groups, unpaired two-tailed Student’s *t*-tests were used. A *p*-value < 0.05 was considered statistically significant. Significance levels are indicated as follows: *p* < 0.05 (*)*, p* < 0.01 (**)*, p* < 0.001 (***).

## Data Availability

The original contributions presented in the study are included in the article; further inquiries can be directed to the corresponding author.

## Supplementary Materials

The following supporting information can be downloaded at https://www.mdpi.com/article/10.3390/antibiotics14070640/s1. Table S1: MICs of Pyf A and antibiotics used clinically against “ESKAPE” pathogens form clinical isolation. Table S2: Antibiotic susceptibility testing of clinical CRKP isolates. Figure S1 Antibiotic susceptibility disk diffusion assay of clinical CRKP isolates. Figure S2: The disruption of mature biofilms following treatment with Pyf A, AgNO_3_, or their combination. Figure S3: CLSM visualization of Live/Dead staining of mature biofilms. Figure S4: Propidium iodide (PI) fluorescence intensity of *K. pneumoniae* strain. Figure S5: Representative hematoxylin and eosin (H&E) and Masson’s trichrome staining of skin tissues. Figure S6: Macroscopic observation of the catheter-associated biofilm infection model.
