# Supplementary material for: Dual Redox Targeting by Pyrroloformamide A and Silver Ions Enhances Antibacterial and Anti-Biofilm Activity Against Carbapenem-Resistant Klebsiella pneumoniae
Source: Antibiotics (Basel). 2025 Jun 23;14(7):640. doi: 10.3390/antibiotics14070640 (PMC12291900; doi:10.3390/antibiotics14070640)

Supplementary Information

Dual Redox Targeting by Pyrroloformamide A and Silver Ions Enhances Antibacterial and Anti-Biofilm Activity Against Carbapenem-resistant *Klebsiella pneumoniae*

Enhe Bai[1], Qingwen Tan[1], Yi Xiong[1], Jianghui Yao[1], Yanwen Duan[* 1,2,3], Yong Huang[* 1,4]

[1]Xiangya International Academy of Translational Medicine, Central South University, Changsha 410013, China

[2]Hunan Engineering Research Center of Combinatorial Biosynthesis and Natural Product Drug Discovery, Changsha 410013, China

[3]National Engineering Research Center of Combinatorial Biosynthesis for Drug Discovery, Changsha 410013, China;

[4]Hefei Comprehensive National Science Center, Institute of Health and Medicine, Hefei 230093, China

[*]To whom correspondents should be addressed: Dr. Yong Hang, Central South University, Tongzipo Road，#172，Yuelu District，Changsha, Hunan 410013，China. Tel：(86) 731 8265 0539；Fax：(86) 731 8265 0551；Email：jonghuang@ihm.ac.cn, Prof. Yanwen Duan, Email: ywduan66@sina.com.

This file includes:

**Table S1**: MICs of Pyf A and clinically-used antibiotics against ESKAPE pathogens.	Page 1
**Table S2:** Antibiotic susceptibility results of clinical CRKP isolates.	Page 2
**Figure S1:** Antibiotic susceptibility disk diffusion assay of clinical CRKP isolates.	Page 3
**Figure S2**: Bright-field microscopy images of CRKP biofilms	Page 4
**Figure S3:** CLSM visualization of Live/Dead staining of mature biofilms.	Page 5
**Figure S4:** Propidium iodide fluorescence intensity of KP113.	Page 6
**Figure S5:** H&E and Masson's trichrome staining of skin tissues.	Page7
**Figure S6:** Macroscopic observation of the catheter-associated biofilm infection model.	Page 8

Table S1. MICs of Pyf A and clinically-used antibiotics against ESKAPE pathogens.

Species	Strains	MIC (μg/mL)						
		Pyf A	Pyf B	Norfloxacin	Ampicillin	Imipenem	Polymyxin B	Vancomycin
K. pneumoniae	130-020-081	4.0	4.0	16.0	>128	16.0	64.0	–
	130-020-110	4.0	2.0	64.0	>128	16.0	64.0	–
	130-020-113	4.0	4.0	64.0	>128	64.0	64.0	–
A. baumannii	130-020-066	2.0	2.0	16.0	64.0	4.0	2.0	–
E. cloacae	130-020-039	1.0	0.5	4.0	64.0	0.5	4.0	–
P. aeruginosa	130-020-028	8.0	4.0	64.0	32.0	16.0	16.0	–
E. faecium	130-020-017	2.0	2.0	16.0	64.0	1.0	2.0	–
S. aureus	ATCC 29213	0.5	0.5	1.0	1.0	1.0	4.0	1.0
	130-020-116 (MRSA)	0.5	0.5	2.0	2.0	8.0	1.0	2.0

Table S2. Antibiotic susceptibility results of clinical CRKP isolates.

	KP081	*KP110*	*KP113*
Ampicillin	R	R	R
Gentamycin	S	R	R
Norfloxacin	S	R	R
Imipenem	S	S	R
Polymyxin B	—※	—※	—※

S-sensitive, I-intermediate, R-resistant

According to the European Committee on Antimicrobial Susceptibility Testing (EUCAST), the drug susceptibility of bacteria is defined as follows:

Klebsiella pneumoniae zone diameter breakpoints (mm):

- Ampicillin: $R < 14$, $S \geq 14$
- Gentamicin: $R < 17$, $S \geq 17$
- Norfloxacin: $R < 22$, $S \geq 22$
- Imipenem: $R < 19$, $S \geq 22$

Note: ※Polymyxin has no defined zone diameter breakpoint for determining drug susceptibility.

Figure S1 Antibiotic susceptibility testing of clinical CRKP isolates. Disk diffusion assay was performed on three clinical carbapenem-resistant *Klebsiella pneumoniae* (CRKP) isolates (KP081, KP110, and KP113) using polymyxin B, imipenem, norfloxacin, gentamycin, and ampicillin. Saline served as a negative control. The assay was conducted and interpreted according to EUCAST guidelines.

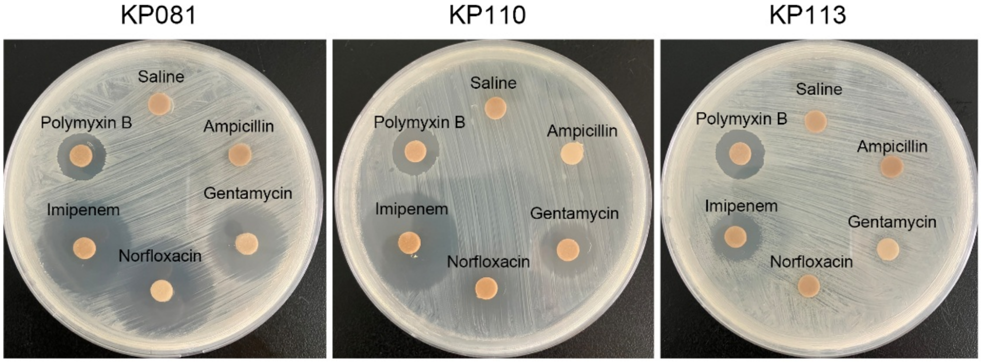

Figure S2. Bright-field microscopy images of CRKP biofilms. Bright-field microscopy images illustrated the disruption of mature biofilms following treatment with Pyf A, $AgNO_3$, or their combination. Images were acquired at 50× magnification, with a scale bar representing 100 μm.

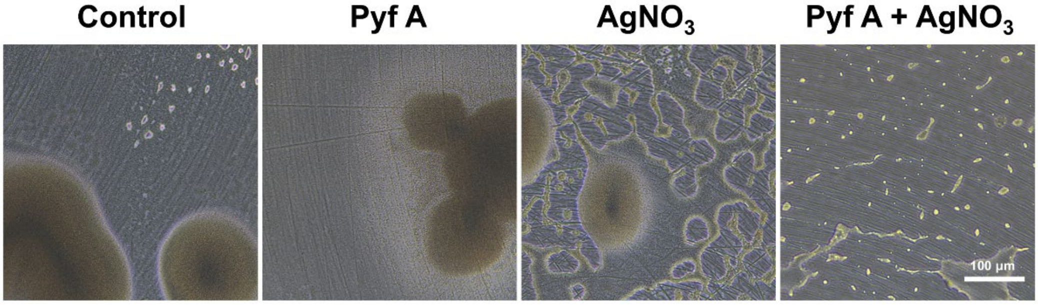

Figure S3. CLSM visualization of Live/Dead staining of mature biofilms. Confocal laser scanning microscopy (CLSM) images showing the bactericidal effects of Pyf A, $AgNO_3$, and their combination on mature biofilms, assessed by live/dead cell staining. Images were captured at 100× magnification with a scale bar of 200 μm. Three-dimensional reconstruction and visualization were performed using Image-Pro Plus.

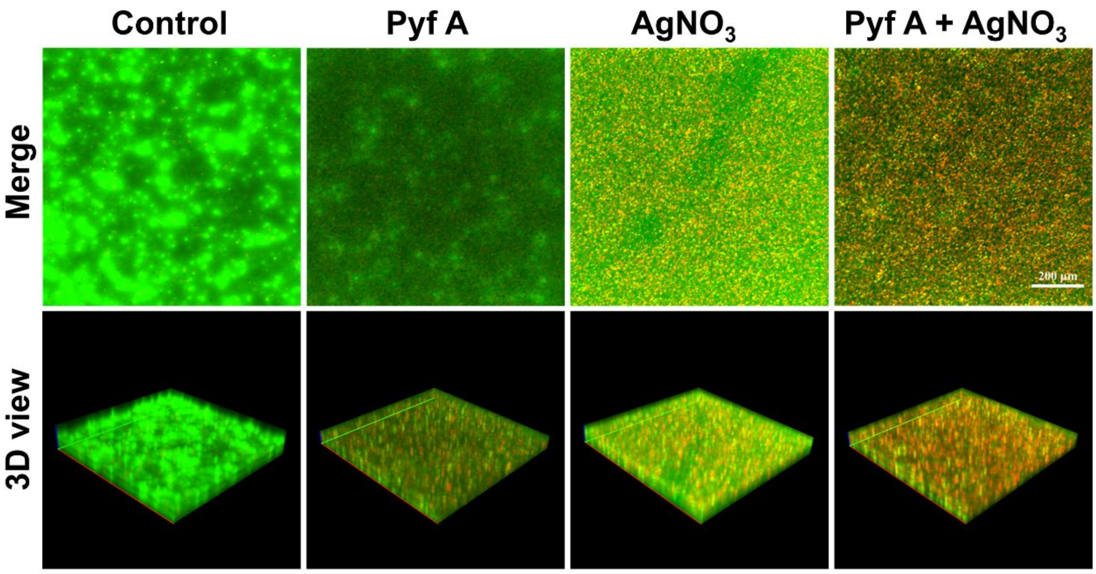

Figure S4. Propidium iodide fluorescence intensity of KP113. PI staining assay was used to assess the membrane permeability of *Klebsiella pneumoniae* strain (KP113). Data represent mean ± SD (n = 3).

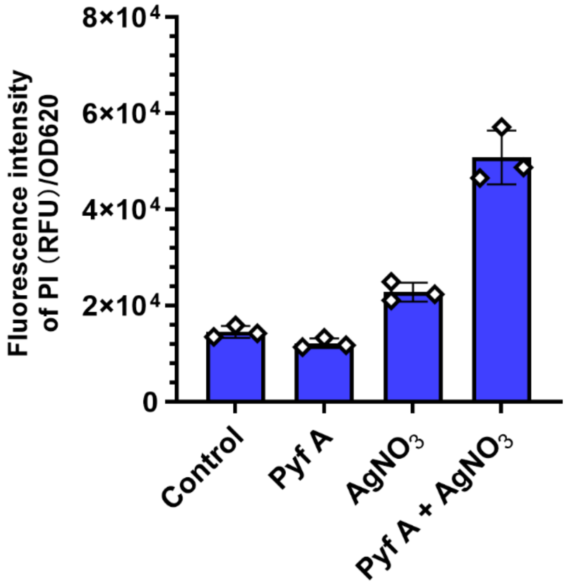

Figure S5. Representative hematoxylin and eosin (H&E) and Masson's trichrome staining of skin tissues from the murine wound infection model. Images were acquired at 50× magnification; scale bar represents 200 μm.

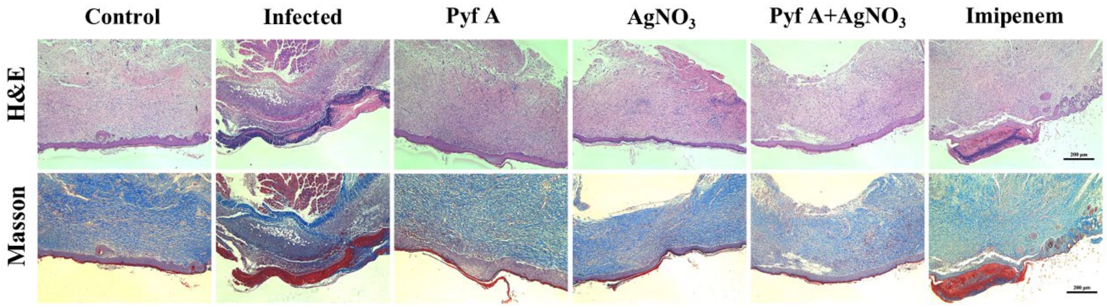

Figure S6. Macroscopic observation of the catheter-associated biofilm infection model, including anatomical assessment of the catheter and surrounding tissue responses.

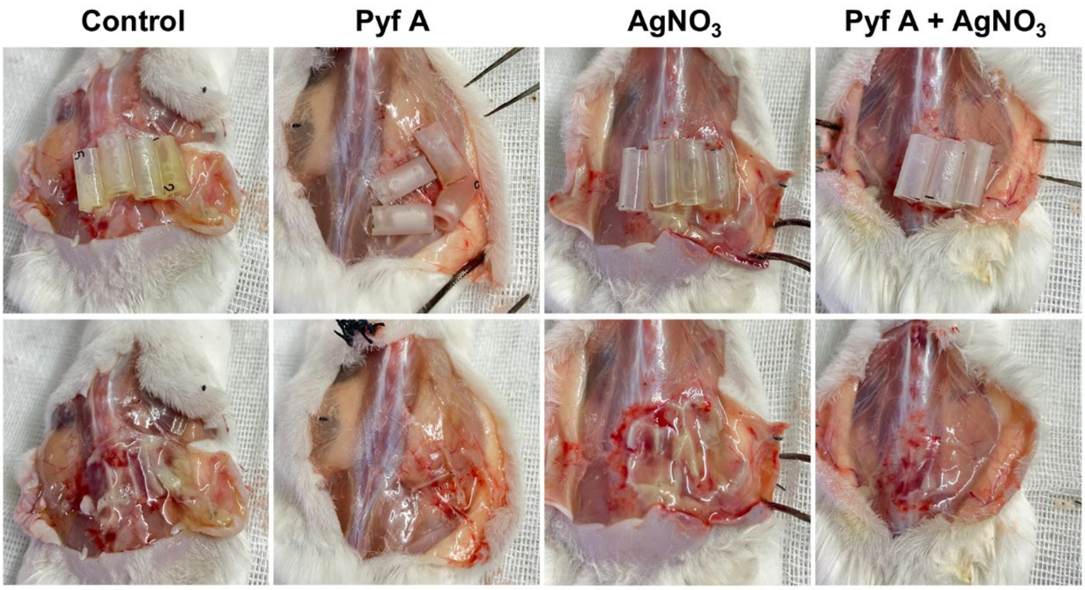

Supplement: Supplementary file 1 [file antibiotics-14-00640-s001.zip › antibiotics-3703316-supplementary.pdf]
